# Untangling Peripheral Sympathetic Neurocircuits

**DOI:** 10.3389/fcvm.2022.842656

**Published:** 2022-02-10

**Authors:** Courtney Clyburn, Michael C. Andresen, Susan L. Ingram, Beth A. Habecker

**Affiliations:** ^1^Department of Chemical Physiology and Biochemistry, Oregon Health and Science University, Portland, OR, United States; ^2^Department of Neurological Surgery, Oregon Health and Science University, Portland, OR, United States

**Keywords:** sympathetic ganglia, neurocircuits, synaptic inputs, co-transmission, collaterals

## Abstract

The sympathetic nervous system plays a critical role in regulating many autonomic functions, including cardiac rhythm. The postganglionic neurons in the sympathetic chain ganglia are essential components that relay sympathetic signals to target tissues and disruption of their activity leads to poor health outcomes. Despite this importance, the neurocircuitry within sympathetic ganglia is poorly understood. Canonically, postganglionic sympathetic neurons are thought to simply be activated by monosynaptic inputs from preganglionic cholinergic neurons of the intermediolateral cell columns of the spinal cord. Early electrophysiological studies of sympathetic ganglia where the peripheral nerve trunks were electrically stimulated identified excitatory cholinergic synaptic events in addition to retrograde action potentials, leading some to speculate that excitatory collateral projections are present. However, this seemed unlikely since sympathetic postganglionic neurons were known to synthesize and release norepinephrine and expression of dual neurochemical phenotypes had not been well recognized. *In vitro* studies clearly established the capacity of cultured sympathetic neurons to express and release acetylcholine *and* norepinephrine throughout development and even in pathophysiological conditions. Given this insight, we believe that the canonical view of ganglionic transmission needs to be reevaluated and may provide a mechanistic understanding of autonomic imbalance in disease. Further studies likely will require genetic models manipulating neurochemical phenotypes within sympathetic ganglia to resolve the function of cholinergic collateral projections between postganglionic neurons. In this perspective article, we will discuss the evidence for collateral projections in sympathetic ganglia, determine if current laboratory techniques could address these questions, and discuss potential obstacles and caveats.

## Introduction

Technical advances have long driven new insights into the mechanistic basis of neurophysiology. From the identification of the action potential in the nineteenth century (1) to the work of Joseph Erlanger and Herbert Gasser who revolutionized neurophysiological research by developing sensitive oscilloscopes that allowed for the visualization and analysis of nerve impulses previously below the threshold of detection (2, 3). With these technological advancements, researchers set out to decode the complex autonomic signals that regulate visceral functions, including cardiac activity (4–6). Several important hypotheses arose from this work, but were untested due to experimental limitations that existed at the time. These questions include whether or not collateral projections are present between postganglionic neurons in sympathetic ganglia and what neurotransmitters may be involved in sympathetic signaling pathways. Since these first studies, significant advancements in neurophysiological equipment and scientific methods have allowed for substantial progress in understanding the neurophysiology of peripheral sympathetic activity (7–11), but the neurocircuitry within sympathetic ganglia remains poorly understood. The purpose of this article is to review the primary data that support these untested hypotheses, determine if current technology and experimental methods can be used to answer these important questions, and discuss any potential obstacles that may arise.

### Canonical Neurocircuitry of Cervical Sympathetic Ganglia

Several well-written review articles are available that thoroughly summarize our current understanding of the sympathetic neurocircuitry that regulates visceral functions and cardiac activity (12–16). To briefly summarize, sympathetic signals originate in the hypothalamus and brainstem and travel to the preganglionic neurons in the intermediolateral cell column of the spinal cord. These preganglionic neurons send cholinergic projections to postganglionic neurons in the cervical sympathetic chain ganglia. These neurons in the sympathetic ganglia then send noradrenergic projections through the postganglionic nerve trunk to innervate the target tissue. This general organization is conserved across species. However, there is significant biological variability in the anatomy and physiology of the cervical sympathetic ganglia which clouds our understanding of peripheral sympathetic neurocircuits. The cervical sympathetic chain includes the superior, middle, and inferior cervical ganglia and exhibits significant anatomical variability between species, between individuals, and even between the left and right ganglia. In approximately 80% of humans, for example, the inferior cervical ganglion is fused with the first thoracic ganglion to form the cervicothoracic (commonly known as stellate) ganglion. Additionally, the middle cervical ganglion is completely absent in ~20% of the population (17–20). In considering the variability in the sympathetic nervous system in humans and animal models as well as the murkiness that surrounds the functional organization of the cervical sympathetic ganglia, we will consider data from all cervical sympathetic ganglia interchangeably as we discuss peripheral sympathetic neurocircuitry.

### Evidence for Collateral Projections in Sympathetic Ganglia

**Figure 1:**
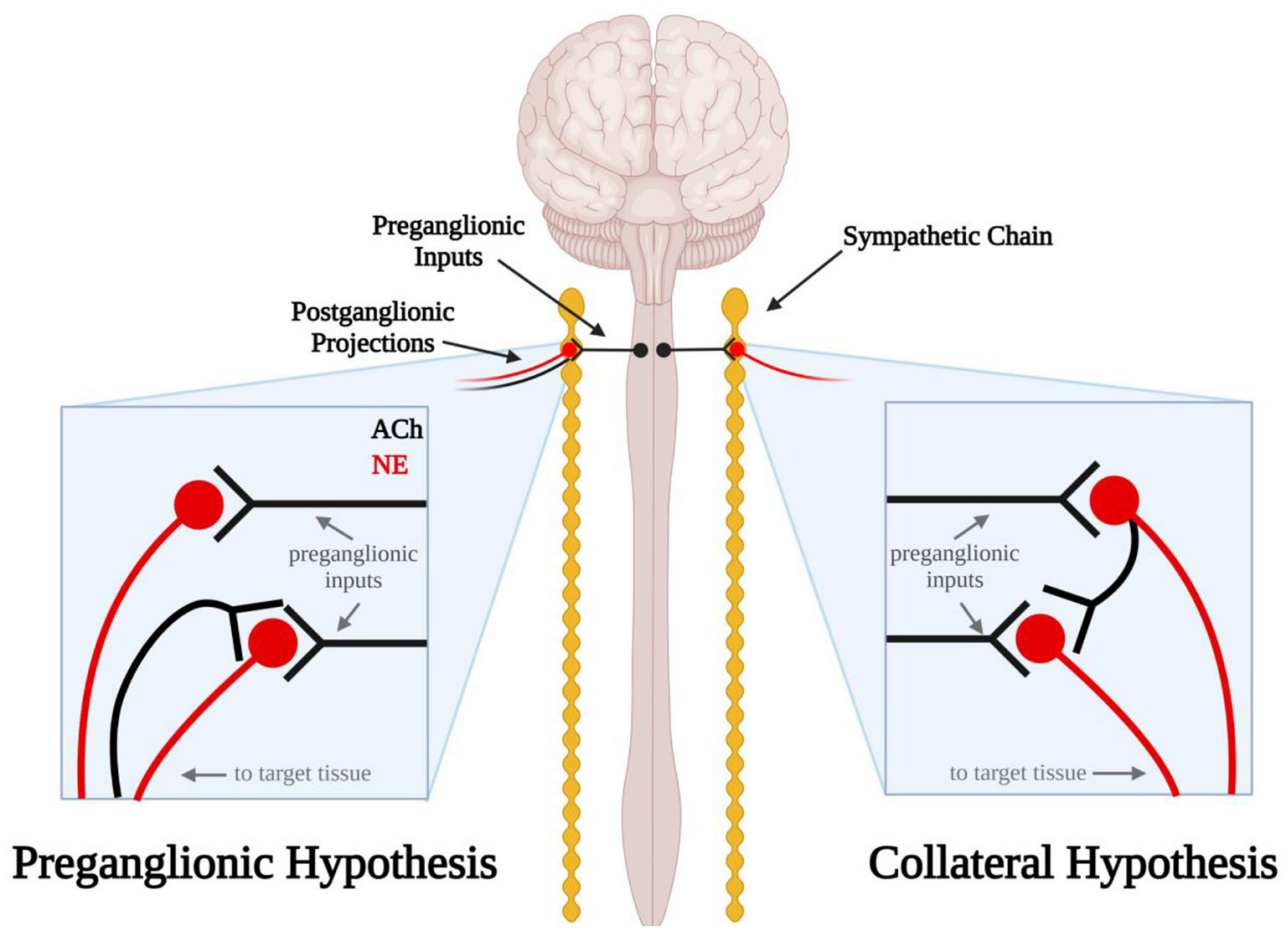
Schematic diagram illustrating the errant preganglionic fiber and collateral hypotheses. In the preganglionic hypothesis (left), mis-routed cholinergic (black) preganglionic fibers travel back up the efferent nerves through which sympathetic axons travel to the target tissue. In the collateral hypothesis (right) postganglionic sympathetic neurons send noradrenergic projections (red) to the target tissue and cholinergic projections (black) to other sympathetic neurons in the sympathetic chain ganglia.

In addition to the ambiguity surrounding the anatomy of the sympathetic ganglia, the functional organization of the sympathetic neurocircuitry within these ganglia remains poorly defined. Current literature usually describes and illustrates the neurocircuitry within cervical sympathetic ganglia as simple monosynaptic connections from cholinergic preganglionic neurons to the noradrenergic postganglionic neurons that innervate visceral targets and the heart (21, 22). However, Erulkar and Woodward (23) made intracellular recordings of postganglionic sympathetic neurons from rabbit superior cervical ganglia (SCG) *in situ* which suggest this neurocircuitry may be more complicated. In these early experiments, they found that stimulation of the external carotid nerve produced single, short latency spikes followed by long-lasting hyperpolarizations (presumably action potentials) in some neurons. In these experiments, electrical stimulation of the postganglionic nerve trunk evoked antidromic, or retrograde, action potentials that traveled up the axon to the cell body in the SCG. Surprisingly, the majority of neurons exhibited an early spike followed by a long-lasting depolarization. This depolarization suggested that stimulation of the postganglionic nerve trunk elicited excitatory synaptic activity within the SCG. Erulkar and Woodward proposed several hypotheses that would account for this phenomenon, but state that the simplest explanation would be that recurrent excitatory collateral fibers from postganglionic axons project to neighboring cells within the SCG (Figure 1). Upon stimulation of the preganglionic nerve trunk, Erulkar and Woodward also observed that a significant proportion of SCG neurons exhibited multi-spike responses which were uncovered at increasing stimulation intensities. While they acknowledge these results may be caused by different populations of preganglionic fibers with different thresholds and conduction velocities that converge onto the same cell, this explanation seems highly unlikely. Instead, they note that the multi-spike responses are consistent with the collateral hypothesis and that the late-arriving impulses may have traveled through another indirect pathway.

Excitatory synaptic events in postganglionic sympathetic neurons following electrical stimulation of the postganglionic nerve trunk have also been observed in the cat, rat and guinea pig (24–26). In 1970, Perri et al. extended the observations by Erulkar and Woodward by investigating the neurotransmitter that may be involved in these synaptic events. Curare, a nicotinic acetylcholine (ACh) receptor antagonist, applied to the ganglionic preparation abolished these synaptic events, indicating they were likely cholinergic in nature (25). If these excitatory events originated from postganglionic collateral projections, this would mean that these sympathetic neurons express ACh *and* norepinephrine (NE). Although we now know that postganglionic sympathetic neurons and other neurons can express a dual neurochemical phenotype (27–31), at the time this was thought to be unlikely. Thus, researchers sought alternative explanations. These included the possibility that errant preganglionic fibers may be running up the postganglionic nerve trunk and forming excitatory synaptic connections with postganglionic neurons (Figure 1) (25). A second possibility raised was that sensory afferent neurons were the source of synaptic excitation. Sympathetic ganglia have long been linked to sensory afferents of the cardiac region of the thoracic viscera (6) but on the bases of such anatomical findings broadly dismissed as only passing through the ganglia on their way to the spinal cord. Thus, sketches commonly depict that a portion of these afferent paths include sympathetic ganglia (e.g., cervical and stellate) (32). However, functional studies related to cardiovascular regulation, for example, indicate that activation of cardiac sensory afferents traveling via the stellate ganglia act to increase blood pressure by augmenting sympathetic motor neuron activity directed to the systemic vasculature and such responses are abolished by severing ganglion connections to the spinal cord (33). Dye tracing and electrical activity recordings indicate that such afferent neurons arise from the dorsal root ganglia and traverse peripheral sympathetic ganglia to their visceral targets (34). Although it seemed unlikely at the time, our current understanding of dual neurochemical phenotypes in sympathetic neurons has persuaded us that further studies are needed to investigate the potential presence of cholinergic collateral projections between postganglionic neurons in sympathetic ganglia.

### Evidence for Dual Neurochemical Phenotypes in Sympathetic Neurons

In 1935, Henry Dale put forth the “one neuron, one neurotransmitter” hypothesis which was later termed as “Dale's Principle” (35, 36) and stated that one neuron was only capable of releasing one neurotransmitter at any one time [reviewed by: (37)]. By the mid-1970s, evidence disputing Dale's Principle began to emerge when Jan and colleagues made intracellular recordings of sympathetic frog neurons and observed slow synaptic potentials which were mediated by the peptide, luteinizing hormone-releasing hormone, in addition to the canonical cholinergic transmission (38, 39). Since these initial studies, co-transmission of several neuromodulators, including ATP and neuropeptide Y (NPY) (40–44), as well as the co-transmission of fast primary neurotransmitters have been well established throughout the central and peripheral nervous system (45–47).

Some of the earliest evidence supporting co-expression of primary neurotransmitters comes from cardiac myocyte—sympathetic neuron co-cultures (48). In 1976, Furshpan et al. used electrophysiological recordings of sympathetic SCG principal neurons cultured with cardiac myocytes from newborn rats and described a population of neurons that secreted ACh *and* NE (48–51). This work sparked a series of studies to determine if sympathetic neurons produced ACh *in vivo* and to identify the differentiation factors that determine the fates of sympathetic neurons (52). Several “cholinergic differentiation factors” are now known to induce ACh expression in noradrenergic sympathetic neurons including leukemia inhibitor factor (LIF), ciliary neurotrophic factor (CNTF), cardiotrophin-1 (CT-1), neurotrophin-3 (NT-3) and glial cell line-derived neurotrophic factor (GDNF) (53–61). While the cholinergic transdifferentiation of postganglionic sympathetic neurons *in vivo* was first thought to be confined to developmental periods, more recent studies have shown that inflammatory cytokines can induce cholinergic transdifferentiation in cardiac disease (30, 62). Early studies have revealed key aspects of the neurochemical phenotype of sympathetic neurons: dual and malleable neurochemical capacity. These studies of cultured neurons indicated that these postganglionic neurons could form synapses between neurons and could display adrenergic/cholinergic dual function (50). In culture, the neurotrophin brain-derived neurotrophic factor (BDNF) rapidly shifted sympathetic neuron release of norepinephrine to ACh indicating the dual neurochemical phenotype of these postganglionic neurons (31). Subsequent work also in cultured neurons supported both segregation as well as plasticity of functional release sites of NE and ACh (63). In light of this evidence, it appears that the cholinergic hypothesis originally proposed by Erulkar and Woodward in 1968 was dismissed prematurely before fully testing the idea. Gaining a clear insight into the neurocircuitry within sympathetic ganglia can provide better understanding of the mechanisms underlying autonomic imbalance in disease and may highlight novel therapeutic targets for cardiac patients.

### Challenges and Potential Caveats

The dual expression of ACh and NE in sympathetic neurons provides support for the collateral hypothesis, but there are still many questions that remain. When dual neurochemical phenotypes were first described, these neurons were generally classified into two mechanistic subtypes, co-release and co-transmission (37, 64). Co-release describes the packaging of multiple neurotransmitters into the same synaptic vesicle so they there are released together and co-transmission originally described different neurotransmitters packaged into different vesicles within the same presynaptic terminal (37, 64). Dale's Principle was updated in 1986 to state that neurons release the same group of neurotransmitters/peptides from all presynaptic terminals (64, 65). However, the collateral hypothesis challenges this view because the primary data indicates that ACh is released from the collateral terminals. The excitatory synaptic events that are observed following stimulation of the postganglionic nerve trunk are completely blocked by the application of nicotinic acetylcholine antagonists (24, 25). In the collateral model, this could suggest the biased expression of ACh and NE at different synapses within the same neuron. Based on the updated version of Dale's hypothesis, we would expect that both ACh and NE would be released from collateral synapses. As adrenergic receptors are G-protein coupled receptors, synaptic release of NE cannot be detected with the electrophysiological techniques used in these previous studies (25). Future experiments could utilize new biosensor technology to determine if NE is also released from collateral projections (66–68). If NE is not released from collateral projections, there are several mechanisms that have been described in the CNS that could explain biased release of different vesicular pools. These include differing release probabilities and altered coupling to presynaptic Ca^2+^ channels (69, 70), “kiss-and-run” release mechanisms, and even neurotransmitter segregation to separate axons (64, 71–74). Studies in cultured sympathetic neurons have observed segregation of the vesicular NE and ACh transporters (VMAT2 and VAChT, respectively) in distinct varicosities, suggesting release of each transmitter is independent and spatially segregated (63). Considering how target-derived factors and cytokines can induce cholinergic transdifferentiation in postganglionic sympathetic neurons, it is conceivable that factors in the microenvironment are involved in determining different neurochemical identities of synaptic terminals in the ganglia and target tissues.

Putative afferent fibers could have an important physiological role in allowing sensory feedback to regulate the activity of postganglionic sympathetic neurons in the event that inputs from the central nervous system are disrupted. This sensory feedback loop would also allow for the fast adaptation of sympathetic neuron activity to rapidly changing stimuli (24). However, the synaptic events that are observed following stimulation of the postganglionic nerve trunk are mediated by acetylcholine and, therefore, unlikely to be mediated by sensory fibers, which utilize glutamatergic signaling. The physiological consequence of putative cholinergic collateral projections is less obvious. Collateral projections between postganglionic sympathetic neurons may also have a significant physiological role in maintaining autonomic control of the heart and other tissues. Excitatory collateral projections between postganglionic sympathetic neurons would increase the probability that these neurons fire synchronously and may amplify preganglionic signals from the spinal cord. Given the importance of neural timing for cardiopulmonary integration, the synchronization of sympathetic inputs could potentially play a critical role in maintaining appropriate cardiac activity. Furthermore, disruption of this synchronization following injury or disease could exacerbate autonomic imbalance. Early studies hypothesized that single spikes in recordings of cardiac sympathetic nerve activity represent synchronized activity of multiple postganglionic neurons (75), but the potential role of collateral synapses in this synchronization is purely speculative.

It was not possible in the past to distinguish preganglionic cholinergic transmission from putative post-ganglionic collateral projections, but transgenic mouse models and genetic tools provide the means to address this issue now. Targeted deletion of ACh production (*chat* gene) or release (*slc18A3* gene) from neurons expressing tyrosine hydroxylase (*th* gene), dopamine beta hydroxylase (*dbh* gene), or norepinephrine transporter (*slc6a2* gene) would remove cholinergic transmission selectively from post-ganglionic neurons within the ganglion. Likewise, targeting expression of channelrhodopsin-2 or other stimulatory optogenetic tools to noradrenergic neurons would allow selective stimulation of those cells, coupled with recordings of resulting synaptic activity. Combining these approaches with the use of genetically encoded calcium sensors would allow testing the functional impact of putative cholinergic collateral transmission *in situ*. Mouse lines to facilitate these studies, which directly address the issue of potential cholinergic collaterals, are available from public repositories. If cholinergic collaterals are an important aspect of transmission by post-ganglionic sympathetic neurons then we would expect them to be present and detectable throughout the sympathetic chain, and lead to changes in transmission to target tissues.

## Discussion

While some of the first studies investigating sympathetic nerve impulses proposed that collateral projections may be present in the sympathetic ganglia, this model was dismissed because of our incomplete understanding of dual neurochemical phenotypes. In hindsight, given our understanding of co-transmission of ACh and NE in postganglionic sympathetic neurons, it is clear that studies should be carried out to clarify the neurocircuitry within the sympathetic ganglia. The postganglionic neurons within sympathetic ganglia are critical components of the neurocircuitry responsible for relaying autonomic signals from the central nervous system to several visceral targets, including the heart. The activity of postganglionic sympathetic neurons is disrupted in many pathologies and sympathetic hyperactivity elevates risk for cardiac arrhythmias and sudden cardiac death and contributes to the development of heart failure. A detailed understanding sympathetic neurocircuitry is needed to understand how peripheral sympathetic activity is disrupted in pathophysiological conditions and to develop novel therapeutic strategies.

## Data Availability Statement

The original contributions presented in the study are included in the article, further inquiries can be directed to the corresponding author.

## Author Contributions

CC, MA, SI, and BH contributed to drafting the work and revising it critically for important intellectual content. All authors approved the final version of the manuscript and agree to be accountable for all aspects of the work. All persons designated as authors qualify for authorship, and all those who qualify for authorship are listed.

## Funding

This work was supported by the National Institutes of Health Grant HL146833.

## Conflict of Interest

The authors declare that the research was conducted in the absence of any commercial or financial relationships that could be construed as a potential conflict of interest.

## Publisher's Note

All claims expressed in this article are solely those of the authors and do not necessarily represent those of their affiliated organizations, or those of the publisher, the editors and the reviewers. Any product that may be evaluated in this article, or claim that may be made by its manufacturer, is not guaranteed or endorsed by the publisher.
